# Suprascapular Nerve Compression Secondary to a Spinoglenoid Ganglion Cyst: A Case Report

**DOI:** 10.7759/cureus.36025

**Published:** 2023-03-11

**Authors:** Rouya Alattas, Omer Brinji, Omar A Batouk

**Affiliations:** 1 Department of Orthopedic Surgery, King Fahad General Hospital, Jeddah, SAU; 2 Department of Orthopedics/Surgery, Ministry of National Guard – Health Affairs, Jeddah, SAU

**Keywords:** infraspinatus, open decompression, shoulder pain, suprascapular nerve compression, spinoglenoid ganglion cyst

## Abstract

Suprascapular nerve entrapment is an uncommon entity; it is usually missed as a differential diagnosis of shoulder pain, especially since the main presentation of this condition in patients is usually non-specific shoulder pain. It is often only considered when the patient presents with weakness and denervation of the supraspinatus and infraspinatus muscles. Diagnosis of spinoglenoid ganglion cysts is usually considered after other causes have been ruled out. They are usually detected on magnetic resonance imaging (MRI); however, this could be delayed and happens only after suprascapular nerve compression has already occurred and the patient’s muscles have atrophied leading to limitations in their function.

Treatment of spinoglenoid ganglion cysts should be tailored to each individual patient. Numerous treatment options are available and can range from conservative management to open decompression. The aim of our study was to highlight the clinical presentation of this condition by describing a case that we have diagnosed and managed at our center. We report a 29-year-old male who presented with dysfunctional left shoulder pain. The patient was diagnosed with a spinoglenoid ganglion cyst, which was treated successfully with open excision. The patient's condition improved following the procedure with a successful return to his daily activities.

## Introduction

Shoulder pain is one of the most common musculoskeletal complaints. It may be caused by multiple pathologies, most commonly rotator cuff pathologies, capsular pathologies, or articular pathologies, and in rare cases, suprascapular nerve compression [1]. The suprascapular nerve contains both motor and sensory functions; it supplies the supraspinatus and infraspinatus muscles, and it carries somatic sensation from the glenohumeral and acromioclavicular joints [1]. Compression of the nerve at or above the suprascapular notch can lead to the denervation of both the supraspinatus and infraspinatus muscles. However, compression at the spinoglenoid notch can lead to isolated infraspinatus muscle denervation.

The imaging of choice for non-localized shoulder pain, and specifically for ganglion cysts and other masses, is magnetic resonance imaging (MRI) as it clearly depicts their location, size, and extent. In addition to MRI, electroneuromyography (EMG) can also be used to aid the diagnosis of suprascapular nerve compression, even though it has a high false-negative rate [2].

The diagnosis of a spinoglenoid ganglion cyst is usually considered after other causes have been ruled out. They are usually detected on an MRI; however, this could be delayed and happens only after suprascapular nerve compression has already occurred and the patient’s muscles have atrophied leading to limitations in their function [1]. Due to the rarity of suprascapular nerve entrapment caused by spinoglenoid ganglion cysts, some researchers have prepared case reports to describe the condition, its clinical features, and its management. However, such reports are scarce, and very few of them have been reported in the 21st century [3].

Our study aims to highlight the various aspects of this condition by discussing a case that we have diagnosed and managed at our center. We aim to provide more insight into the topic, especially regarding the clinical presentation, in order for physicians to be able to diagnose spinoglenoid ganglion cysts before the delayed symptoms of suprascapular nerve entrapment occur.

## Case presentation

A 29-year-old male, who is a smoker (one pack-year) with no comorbidities, was referred to the orthopedic surgery clinic from the primary care center with a complaint of left shoulder pain. The patient complained of dull left shoulder pain of one month's duration. He did not report any radiculopathy or neurological delay. There was no history of recent trauma. The pain was relieved initially by non-steroidal anti-inflammatory drugs (NSAIDs) and dexamethasone.

Upon examination, the findings were normal. There were no signs of skin changes, obvious deformities, muscle wasting, swelling, or any asymmetry of the shoulder girdle. Upon palpation, there was no tenderness and no palpable masses. The patient had normal active and passive shoulder range of motion. However, when testing the rotator cuff muscles, there was weakness in the left infraspinatus muscle, which was manifested by weak external rotation while the arm was adducted. Jobe’s and Gerber’s lift-off tests were negative bilaterally. Moreover, when testing for impingement, Neer’s and Hawkin’s tests were normal. Also, Speed’s and Yergason’s tests were performed to test for the biceps, and both tests were normal as well. Both upper limbs were neurovascularly intact for other nerves and vascularity. To summarize, the physical examination was suggestive of infraspinatus tendonitis.

This patient had been previously seen in the neurosurgery clinic for neck pain, and he had been discharged due to a lack of evidence of cervical pathology from their point of view. Additionally, he had been given a local injection of 80 mg of methylprednisolone in the left shoulder along with bupivacaine hydrochloride; however, the pain did not improve.

**Figure 1:**
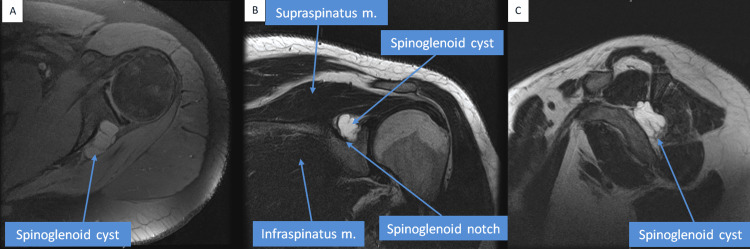
MRI of the patient Selected MRI sections show the presence of a multilobulated cystic-like structure seen extending from the supraspinatus notch reaching anteriorly to the infraspinatus muscle. The lesion measures about 3 x 2 x 3 cm and is consistent with a spinoglenoid ganglion cyst. A. Sagittal view. B. Coronal view. C. Axial view MRI: Magnetic Resonance Imaging

An EMG was done, and it was concluded to be normal. The latency, amplitude, and conduction velocity for the median and ulnar nerves were also normal. Nerve conduction for EMG needling for the deltoid, supraspinatus, and infraspinatus muscles was also normal. Therefore, it was concluded that further assessment was needed. Hence, an MRI of the left shoulder was performed. The MRI report showed the presence of a multilobulated cystic-like structure seen extending from the supraspinatus notch reaching anteriorly to the infraspinatus muscle. The lesion measured about 3 x 2 x 3 cm, and it was consistent with a supraspinous ganglion cyst (Figure 1).

The patient was offered the option of either non-operative or operative treatment. After explaining that the non-operative management includes physiotherapy, and pain medication with or without a local injection, the patient decided to go with the operative management as the pain was affecting his daily life activities. The procedure of choice was open decompression of the suprascapular nerve with excision of the spinoglenoid cyst. The procedure was done successfully with no complications. After excision, the cyst was sent to histopathology to confirm the diagnosis. The histopathological report was consistent with a ganglion cyst.

Description of the procedure

**Figure 2:**
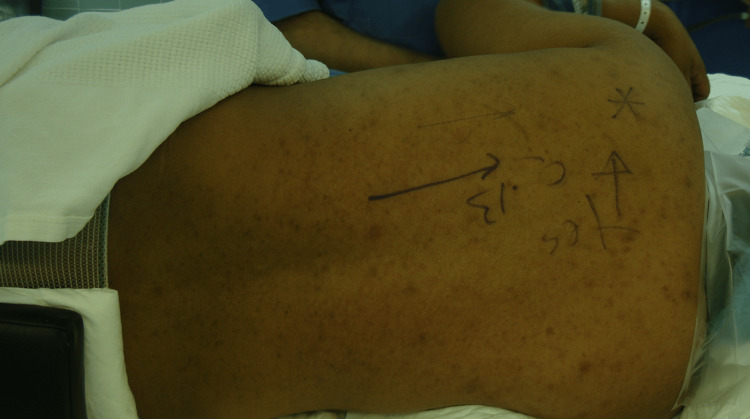
The patient in the semi-prone position

The patient was taken to the operating room. He was put under general anesthesia with endotracheal intubation by the anesthesiologist. Upon induction, the patient received 1 gram of cefazolin. He was positioned in a semi-prone position with left arm support. The left arm was prepped and draped in the usual sterile fashion, freely (Figure 2).

**Figure 3:**
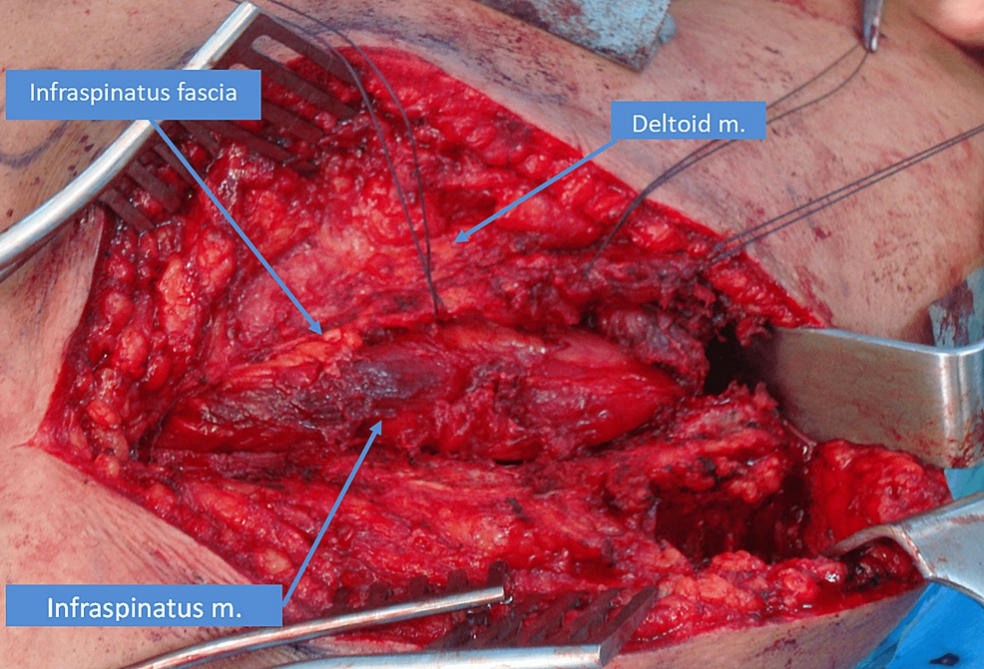
Surgical exposure

A modified Judet posterior skin incision was done, and the subcutaneous tissue incision was cut afterwards using electrocautery. The deltoid insertion was identified and peeled off of the spine of the scapula. The infraspinatus muscle was visualized and peeled off from the infraspinatus fossa subperiosteally. The shoulder joint was identified as well (Figure 3).

**Figure 4:**
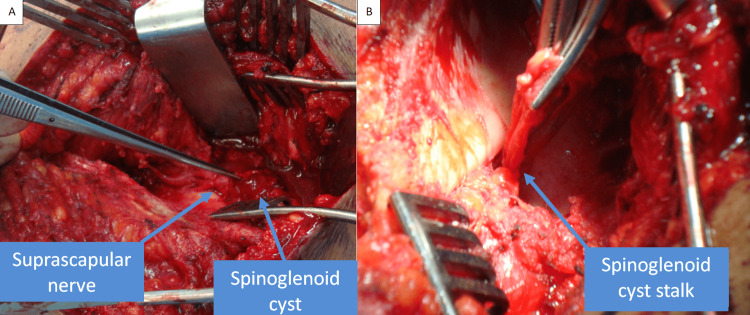
Intraoperative pictures showing the close proximity of the cyst to the suprascapular nerve. Also, the stalk of the cyst is seen, signifying complete excision.

Dissection was started distally until the center was reached. Next, the cyst was identified; it was ruptured during dissection. The suprascapular nerve was identified along with the surrounding vessels, which were protected during the whole procedure. The ganglion was traced down until its neck was seen and until the spinoglenoid notch was reached. Then, it was excised after a couple of sutures were taken to close the neck of the stalk (Figure 4).

**Figure 5:**
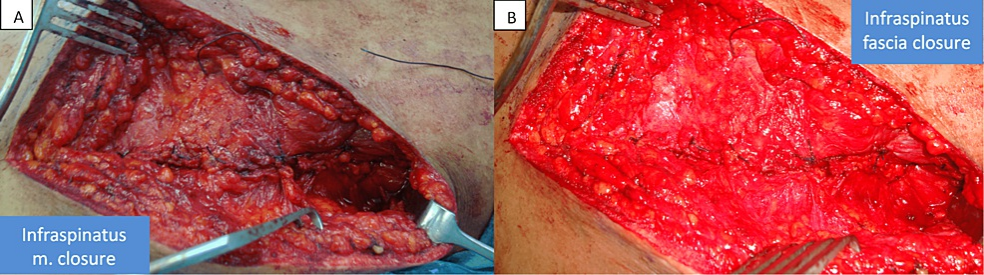
Closure of infraspinatus muscle (A) and fascia (B)

There was no evidence of any pathology inside the glenohumeral joint after an arthrotomy was done. Braided polyester sutures were employed for capsulorrhaphy, and the infraspinatus was repaired using the same suture type. Next, repair of the deltoid to the spine of the scapula was done (Figure 5).

A medium-sized drain was left inside to decrease the risk of hematoma formation. The subcutaneous layer was closed using size 2-0 braided absorbable sutures. The skin was closed with size 3-0 monofilament absorbable sutures, and Steri-Strips (3M, Maplewood, MN) were also applied. Then, a sterile dressing was applied. The patient was brought back to a supine position and extubated successfully, and he was moved to the recovery room in a good condition. He was started on intravenous cefazolin as prophylaxis until drain removal.

Postoperative outcome

Postoperatively, the wound was inspected on day two, and the drain was removed. The patient was on an arm-sling for a period of two weeks. Next, a six-week program of physiotherapy was started, beginning with pendulum exercises. After that, a strengthening program based on a physiotherapy guide for rehabilitation of the shoulder was initiated. He was followed up at two weeks, six weeks, and three months in the outpatient clinic. No complications were noted at follow-ups and the patient regained full painless function after three months of follow-up.

## Discussion

Suprascapular nerve entrapment caused by a spinoglenoid cyst is a rare cause of shoulder pain and functional limitation. Its diagnosis is mostly delayed due to the rarity of this disease. Ultrasound (US) and MRI are crucial in determining the size and location of the lesion and detecting concomitant periarticular shoulder pathology. Moreover, nerve conduction studies (NCS)/EMG studies are beneficial for confirming the diagnosis [4].

Multiple treatment modalities for suprascapular nerve entrapment caused by ganglion cysts have been described in the literature. Conservative treatment including physiotherapy, rest, and analgesia, has mostly been unsuccessful [4]. Nerve decompression is the mainstay of treatment. Options include US/CT-guided aspiration of the cyst as well as arthroscopic or open decompression with or without targeting other associated intraarticular pathologies. Aspiration alone was found to be associated with low morbidity. However, recurrence has been reported, and concurrent periarticular pathologies cannot be addressed with aspiration alone. Arthroscopy for evaluation and treatment of associated labral tears along with aspiration or open decompression has shown to be associated with the least risk of recurrence since labral defects are hypothesized to lead to the formation of a one-way valve that feeds the cyst [5].

Buyukdogan et al. have described a technique in which methylene blue dye was injected preoperatively under US guidance. Next, decompression was done arthroscopically along with addressing intraarticular labral injuries [6]. Open exploration allows for direct visualization along with complete decompression and excision of the cyst. However, if intraarticular lesions are present, they cannot be addressed with this option alone.

The goal of this report is to emphasize the importance of early diagnosis and to highlight the great impact of accurate diagnosis and early treatment on usually active and young patients suffering from suprascapular nerve entrapment. Our patient was symptomatic for more than one month prior to the presentation. He was treated conservatively with analgesia, physiotherapy, and local injections with no improvement. Moreover, he was examined by the neurosurgical team for the possibility of a cervical pathology that might have caused his shoulder pain. NCS/EMG studies were unremarkable at that time. He was eventually diagnosed accurately after undergoing an MRI that clearly demonstrated the cyst. The patient was given the option to either continue conservative treatment or to go for decompression. He chose operative management since the pain was persistent and affecting his daily life. Open surgical excision was done successfully and without any complications. Upon follow-up, the patient was satisfied as he had achieved a successful and painless return to his normal activities.

## Conclusions

Large multilobulated ganglion cysts can lead to suprascapular nerve compression. These cysts can be quite symptomatic and if not treated in a timely fashion, can lead to supraspinatus and/or infraspinatus muscle atrophy and delayed recovery. Open decompression of cysts that are not associated with intraarticular glenohumeral pathology can lead to successful radical treatment with fewer chances of recurrence.
